# Editorial: Special Issue “Optical Signal Processing Technologies for Communication, Computing, and Sensing Applications”

**DOI:** 10.3390/s23052606

**Published:** 2023-02-27

**Authors:** Jiangbing Du, Yang Yue, Jian Zhao, Yange Liu

**Affiliations:** 1 State Key Laboratory of Advanced Optical Communication Systems and Networks, Shanghai Jiao Tong University, Shanghai 200240, China; 2 School of Information and Communications Engineering, Xi’an Jiaotong University, Xi’an 710049, China; 3 School of Precision Instruments and Optoelectronics Engineering, Tianjin University, Tianjin 300072, China; 4 Institute of Modern Optics, Nankai University, Tianjin 300071, China

Optical technology is one of the key technologies that have been widely used for communication, computing and sensing. By utilizing different degrees of freedom for photons, optical signals can be detected and processed in different dimensions, including amplitude, phase, polarization, time, frequency, and spatial mode. Multidimensional signal processing technologies have thus been broadly studied to improve the performance of communication, sensing and even computing systems. Recently, innovative optical signal processing methods and devices have emerged to serve those needs driven by applications including but not limited to optical fiber transmission, supercontinuum generation, phase conjugation, free space optical communication, optical beamforming, photonic integration, fiber amplification, pose estimation and so on.

This Special Issue aims to explore those emerging and enabling technologies of signal processing methods and devices for optical communication, optical computing, and optical sensing. This Special Issue consists of two review papers, one communication, and seven articles.

As for optical signal processing in fiber communications, Y. Zhu et al. presented a proof-of-concept study of stimulated Brillouin scattering (SBS)-induced nonlinear distortion for 10 Gbaud and 28 Gbaud SSB 16QAM transmission over 80 km standard single mode fiber (SSMF) based on a Kramers–Kronig receiver with a significantly reduced bit error rate [1]. M. Tan et al. compared the transmission performances of 600 Gbit/s PM-64QAM WDM signals over 75.6 km of single-mode fiber (SMF) using EDFA, discrete Raman, hybrid Raman/EDFA, and first-order or second-order (dual-order) distributed Raman amplifiers [2]. They also reviewed and studied the designs of distributed Raman amplifiers with respect to nonlinear compensation and bandwidth extension in a mid-link optical phase conjugation system [3,4].

As for optical signal processing for sensing applications, J. Yang et al. designed a silica-cladded Germania-doped ring-core fiber (RCF) that supports orbital angular momentum (OAM) modes. By optimizing the fiber structure parameters, a beyond-two-octave supercontinuum spectrum of the OAM_1,1_ mode can be generated [5]. Regarding 3D human pose estimation, T. Xu et al. reviewed and summarized the recent development on the point cloud-based pose estimation of the human body [6]. The challenges involved and problems to be solved in future studies have also been discussed.

For free space and space division multiplexing applications, C. Álvarez-Roa et al. investigated the application of free space optical (FSO) communications, energy harvesting, and unmanned aerial vehicles (UAVs) as key technology enablers of a cost-efficient backhaul/fronthaul framework for 5G and beyond (5G+) networks [7]. Y. Duan et al. presented a low-complexity robust adaptive beamforming (RAB) method based on an interference-noise covariance matrix (INCM) reconstruction and SOI SV estimation [8]. L. M. Torres et al. studied the linear multiple-input multiple-output (MIMO) receiver designed to optimize the minimum mean square error (MMSE) for a coherent SDM optical communication system, without previous assumptions on receiver oversampling or analog front-end realizations [9].

For the photonic integrated optical signal processing applications, Z. Wang et al. demonstrated a machine learning-based method for agile dispersion engineering of integrated photonic waveguide using a horizontal double-slot structure [10]. Agile dispersion shapes, including broadband low dispersion, constant dispersion and slope-maintained linear dispersion, can be obtained efficiently with high precision.

The Special Issue of optical signal processing only covers few aspects of the powerful and attractive capabilities of optics. Enabling methods, materials, devices, chips and systems for optical signal processing are emerging every day. Optical signal processing powered next-generation communication, computing and sensing can be highly expected.
